# Study of Nightingale’s nursing professionalism: a survey of nurses and nursing students in China

**DOI:** 10.1186/s12912-022-00894-2

**Published:** 2022-05-16

**Authors:** Qian Wang, Chen Wang, Dan Luo, Jin Li, Zhiguang Duan

**Affiliations:** 1 College of Nursing, Shanxi Medical University, Taiyuan, 030001 Shanxi China; 2 Shanxi Medical University, No.56 Xinjian South Road, Taiyuan, 030001 Shanxi China

**Keywords:** Nightingale’s nursing professionalism, Factor analysis, Nurses, Nursing students

## Abstract

**Background:**

Nursing professionalism is highly significant to the development of nursing. Florence Nightingale was the founder and leader of modern nursing, and Nightingale’s nursing professionalism has a significant impact on nursing in China and all over the world. In the new era, a new understanding of Nightingale’s nursing professionalism should be developed, and its positive role in nursing reshaped.

**Methods:**

A total of 1,557 nurses and nursing students from 27 provincial administrative regions of China were surveyed using a customized questionnaire. Their recognitions of Nightingale’s nursing professionalism were evaluated based on scores, and statistical differences between and within the groups were analyzed using an Analysis of Variance (ANOVA). The elements of Nightingale’s nursing professionalism were extracted by the exploratory factor method and a principal component analysis.

**Results:**

The Cronbach’s α of the questionnaire was 0.965, and the two groups did not differ significantly (*p* > 0.05). Based on the standard that the cumulative contribution rate of common factor should be > 85%, three common factors of Nightingale’s nursing professionalism, including professional emotion, professional ability and professional ethics, were extracted based on the Scree plot.

**Conclusions:**

This study aimed to explore the connotation of Nightingale’s nursing professionalism. Our survey indicates that Nightingale’s nursing professionalism is highly recognized by nurses and nursing students in China. Its connotation includes professional emotion, ability and ethics. Nursing education and nursing management should fully utilize the leading role of Nightingale and guide the professional behaviors of nurses to be consistent with Nightingale’s nursing professionalism, thus, improving the degree of nurse professionalism.

## Background

2020 marks the 200th anniversary of the birth of Florence Nightingale, who is the founder of modern nursing. Since she rescued nursing from the ‘sludge’, nurses have gradually taken root in healthcare and have been playing an increasingly important role all over the world. Nurses have been irreplaceable during the COVID-19 outbreak. The World Health Organization (WHO) announced 2020 ss the International Year of Nurse and Midwife to recognize the important contributions of these two communities to global healthcare. All these pronouncements are based on nurse professionalism, which is a key factor in the competence of nurses.

Nevertheless, historians, sociologists and nurses are attempting to determine whether professionalism is still present in this profession [1]. For a long time, nurses have been described as mere executors of doctors’ orders, while their professional skills have not been recognized. Nursing knowledge and skills in nursing science that have accumulated over a long period have been ignored. This phenomenon has been repeated in the media and received much exposure. In other words, most bedside nursing and clinical skills are viewed as byproducts of medical knowledge and judgment. This stereotype results in the belief that nursing requires dedication and following instructions instead of knowledge and skills. Even worse, this stereotype has been strengthened by some practical behaviors of nurses to meet the expected image of public [2].

Indeed, nursing has been developing as a science since the establishment of modern nursing education. Nursing professionalism has existed since the foundation of modern nursing, and it has been continuously developed and improved. In China, it is also known as “Nightingale’s Spirit”. However, a deviation in understanding the connotation of Nightingale’s nursing professionalism has been observed. For example, most people in China believe that Nightingale’s nursing professionalism refers to dedication and sacrifice and that nursing involves serving as a reputable caregiver. This may be related to the internationally renowned Nightingale Medal that emphasizes sacrifice and dedication [3, 4]. Owing to Florence Nightingale's far-reaching influence on modern nursing and her irreplaceable role, most people picture Florence Nightingale whenever nurses are mentioned.

However, in 2003, the American newspaper the Washington Post published an article entitled *Good night, Florence: with nursing in crisis, some say it's time to retire Nightingale as a symbol* [5]. Is Florence Nightingale no longer the flag of caregivers? Is Nightingale’s nursing professionalism out of date? Substantial efforts have been invested in studies of nursing professionalism. Researchers [6–8] have investigated and practiced the professional scale from different perspectives, which provide models and methods to measure nurse professionalism. Nevertheless, the recognition of nursing professionalism by nurses should be clarified, and nurses themselves should rid themselves of this stereotype first, since nursing professionalism reflects the attitude of nurses toward their work and serves as a guide for their behaviors. Previous studies have shown that the job satisfaction of nurses is proportional to the professionalism level of nursing [9]. A study on the internet image of nurses indicates that nurses are typically described as workers who are responsible for comforting patients and recording data [10]. Florence Nightingale has been described as a lantern goddess, which does not reflect the professionalism of nursing. This would appear to have a negative impact on the development of nursing professionalism. In particular, the public tends to ignore the professional value of nurses, resulting in negative influences on the recruitment of nursing staff, allocation of resources and awareness of nursing professionalism. It has been demonstrated that the simple view of nursing plays a dominant role if the image of nursing is based only on the stereotype [11]. Therefore, a change in the perception of nursing may be as necessary today as in the Nightingale Era. Additionally, it is necessary to review the concept of nursing professionalism to provide a conceptual definition for the twenty-first century. This is particularly necessary since professionalism is a complex and multi-dimensional structure, which differs with historical and cultural backgrounds [12]. Similarly, Nightingale’s nursing professionalism should currently be re-examined to explore its enduring essence in nursing. In this study, the current impacts of Nightingale's professional values on nurses were investigated with Nightingale’s nursing professionalism as the starting point. Recognition of the connotation of Nightingale's nursing professionalism by nurses and nursing students in China was investigated.

## Methods

### Ethics

The objectives, methods, investigation measures and application of the results of this study were explained to participants and approved by the Ethics Committee of the Nursing College of Shanxi Medical University (Taiyuan, China). Participants of online surveys were assured of anonymity and voluntary participation. All the data acquired was used for this study only, and all the information provided was kept confidential.

### Design, population and samples

In this study, a cross-sectional survey design was employed and supplemented by qualitative research to determine the connotation of Nightingale’s nursing professionalism. Moreover, the recognition of its contents by nurses and nursing students in China was investigated to clarify the leading role of Nightingale’s nursing professionalism in contemporary nursing. Online questionnaires were allocated to the target population using convenience and snowball sampling. The inclusion criteria of the survey respondents were all types of nurses and undergraduate or graduate nursing students who were willing to participate. A total of 1,557 participants included nurses at different levels of hospitals and undergraduates and postgraduates who majored in nursing in 27 provincial administrative regions of China.

### Procedures

Initial item design: The items about the connotation of Nightingale’s nursing professionalism were developed based on relevant historical documents and Nightingale’s works, such as hospital and nursing notes. The questionnaire included two parts: general information and the connotation of Nightingale’s nursing professionalism. A total of 11 questions surveyed examined general information (Table 1), while 25 questions surveyed the connotation of Nightingale’s nursing professionalism.

**Table 1:** Statistics of the general conditions of samples (*n* = 1,557)

Variables		Category	Sample size (%)	Total
Gender		M	95(6.10)	1557
		F	1462(93.90)	
Non-student participants	Position	Nurse	426(70.30)	606
		Nursing manager	73(4.69)	
		Teacher	107(6.87)	
	Education	Technical secondary school and junior college	87(14.36)	606
		Undergraduate	420(69.31)	
		Postgraduate	99(16.34)	
	Professional title	Primary	281(46.37)	606
		Intermediate	237(39.11)	
		Deputy senior	74(12.21)	
		Senior	14(2.31)	
	Age	< 35	318(52.47)	606
		35–49	242(39.93)	
		≥ 50	46(7.59)	
	Length of service	< 15	394(65.02)	606
		15–29	174(28.71)	
		≥ 30	38(6.27)	
Student participants	Postgraduate	First-year	86(9.04)	951
		Second-year	17(1.79)	
		Third-year	33(3.47)	
	Undergraduate	Freshmen	269(28.29)	
		Sophomore	162(17.03)	
		Junior	209(21.98)	
		Senior	175(18.40)	

Questionnaire revision: Ten experts were invited to fill in the questionnaire through face-to-face discussion. Feedback was collected and discussed. Items with expert recognition rates < 20% were modified or deleted [13], resulting in a final total of 14 items on the connotation of Nightingale’s nursing professionalism survey (Table 2)**.** All the items were assessed using a significance level from 1 to 10 based on the 10-level matrix scale. One and 10 refer to the least and most important items, respectively.

**Table 2:** Scores of items about the connotation of Nightingale’s nursing spirit by different populations and statistical analyses (X ± S)

Item	Nurses	Teachers	Nursing management	Undergraduates	Postgraduates	Total	F value	*P* value
Dedication	8.33 ± 2.27	8.19 ± 2.52	8.50 ± 2.27	8.28 ± 2.13	8.47 ± 2.17	8.31 ± 2.21	0.439	0.780
Sympathy	8.51 ± 2.11	8.28 ± 2.48	8.62 ± 2.38	8.25 ± 2.14	8.59 ± 2.00	8.37 ± 2.16	1.721	0.143
Keen insight	8.40 ± 2.08	8.11 ± 2.45	8.63 ± 2.09	8.14 ± 2.16	8.44 ± 2.03	8.26 ± 2.15	2.041	0.086
Professional judgement	8.57 ± 1.96	8.46 ± 2.26	8.70 ± 2.04	8.29 ± 2.18	8.58 ± 1.99	8.42 ± 2.11	1.848	0.117
Professional care	8.59 ± 1.97	8.49 ± 2.34	8.81 ± 1.97	8.36 ± 2.20	8.54 ± 2.05	8.47 ± 2.13	1.347	0.250
Leadership	7.69 ± 2.32	7.31 ± 2.55	8.09 ± 2.16	7.72 ± 2.31	7.93 ± 2.15	7.72 ± 2.31	1.605	0.171
Innovation	7.83 ± 2.23	7.43 ± 2.38	7.43 ± 2.27	7.75 ± 2.31	7.92 ± 2.24	7.75 ± 2.29	1.233	0.295
Teamwork	8.59 ± 2.04	8.4 ± 2.25	8.54 ± 2.17	8.4 ± 2.08	8.42 ± 2.05	8.46 ± 2.08	0.684	0.603
Respect for life	9.09 ± 1.84	9.11 ± 1.98	9.14 ± 2.06	9.01 ± 1.95	8.98 ± 1.91	9.04 ± 1.92	0.225	0.924
Soothe	8.80 ± 1.93	8.65 ± 2.16	8.74 ± 2.13	8.58 ± 2.05	8.70 ± 1.94	8.67 ± 2.02	0.880	0.475
Help	8.88 ± 1.85	8.58 ± 2.07	8.76 ± 2.12	8.68 ± 1.98	8.61 ± 1.98	8.72 ± 1.96	0.995	0.409
Exquisite technique	8.57 ± 1.91	8.34 ± 2.25	8.28 ± 2.14	8.33 ± 2.19	8.48 ± 2.04	8.41 ± 2.10	1.024	0.394
Love and dedication	8.93 ± 1.89	8.69 ± 2.07	9.07 ± 2.10	8.69 ± 2.00	8.75 ± 1.92	8.78 ± 1.98	1.520	0.194
Being prudent and responsible	8.87 ± 1.92	8.71 ± 1.99	8.98 ± 2.09	8.72 ± 2.00	8.87 ± 1.89	8.79 ± 1.97	0.678	0.607
Total	119.57 ± 25.44	116.68 ± 27.25	120.23 ± 26.56	117.14 ± 26.75	119.21 ± 25.52	118.1 ± 26.31	0.861	0.487

Inclusion criteria for experts: Experts who had been engaged in nursing-related majors for > 10 years and have professional titles, such as associate professor (or their equivalent), were invited. They are familiar with Nightingale’s deeds and nursing values, and their participation was voluntary.

Composition of experts: The experts included one Nightingale Medalist, two nursing management experts, two nursing student management experts, two nursing education experts, one senior nurse, one clinician and one questionnaire expert. The diversity of fields of the experts enabled the examination of Nightingale’s nursing professionalism from a comprehensive and broad perspective to remove the cognitive bias caused by professional limitations.

Pre-survey: Fifty questionnaires were distributed to nursing students of Shanxi Medical University and nurses of its affiliated Hospital.

Content validity: Six nursing experts were invited to repeatedly check the items during the preparation. In particular, the items should represent Nightingale’s nursing professionalism and be suitable for judging the professionalism of nursing. Additionally, there should be no differences between the items and hypothetical concepts. All the experts believed that the items were representative and involved nursing professionalism.

Confidence: The internal consistency was examined using Cronbach’s α, and the confidence level obtained was 0.965.

Estimation of sample size:

$$n\, = \,\left( {\frac{{Z_{1 - a/2} }}{\delta }} \right)^{2} \times p \times (1 - p)$$

Where n refers to the sample size, and α refers to the significance level. When 0.05 is selected for a bilateral test, Z1-α/2 = 1.96. δ refers to the tolerable error, which is 0.05 in this study. *p* refers to the sample rate, which was 36% in this study owing to unknown reasons. In the qualitative interview with 11 Nightingale Medalists at the early stage of this study, 36% of the survey results of Nightingale as a professional example was taken as the core index.

As observed, each group contained no less than 354 participants. In practice, 424 participants (20% higher than 354) were involved in each group considering the sample effective rate and the sample diversity. In this study, nurses and nursing students were the main communities, and their total sample size was no less than 848. Questionnaires were distributed in February and March 2021 via Questionnaire Star, an online survey platform,. A total of 1,663 questionnaires were collected, with 1,557 of them valid (effective rate = 93.62%). A total of 106 items on the questionnaire were excluded for missing items. The two survey groups met the minimum sample size requirements (Table 1).

### Statistical analysis

SPSS 22.0 (IBM, Inc., Armonk, NY, USA) was utilized to statistically describe (X ± S) the characteristic indices of the participants, including professional title, age, length of service, grades of the students, and their scores for each item by each population. Statistical differences between and within groups were analyzed using an Analysis of Variance (**ANOVA)**. Statistical significance is indicated by *p* < 0.05. The connotation of Nightingale’s nursing professionalism was analyzed using exploratory factor and principal component analyses. A varimax orthogonal rotation was executed, and the common factor was extracted by a cumulative contribution rate > 85% based on the Scree plot [14].

## Results

### Sociodemographic characteristics of participants

Among the participants, 93.9% were female, and 6.1% were male. The lengths of service of nurses and teachers ranged from 0.5 to 41 years, and their ages ranged from 20 to 61 years. Among the in-service nurses, 69.31% were undergraduates and 16.34% were postgraduates.

### Statistical analysis of variable scoring in different populations

Table 2 summarizes the representative scores by different populations and the statistical analysis of the results. As observed, the average scores of all items exceeded 7, and no statistical differences were observed between and within groups (*P* > 0.05).

### Exploratory factor analysis of Nightingale’s nursing professionalism

Factor analysis categorizes original variables based on the correlation, so that the correlations of variables in the same group are high, while the correlations of variables in different groups are low. Each group of variables is represented by an unobservable hypothetical variable, which is regarded as the common factor. These common factors can reflect the main information of original variables. In other words, most information provided by the original variables are represented by comprehensive indicators. First, 14 indicators in the questionnaire were assigned, and a factor correlation analysis was applied for each variable in SPSS 22.0.

**Fig. 1:**
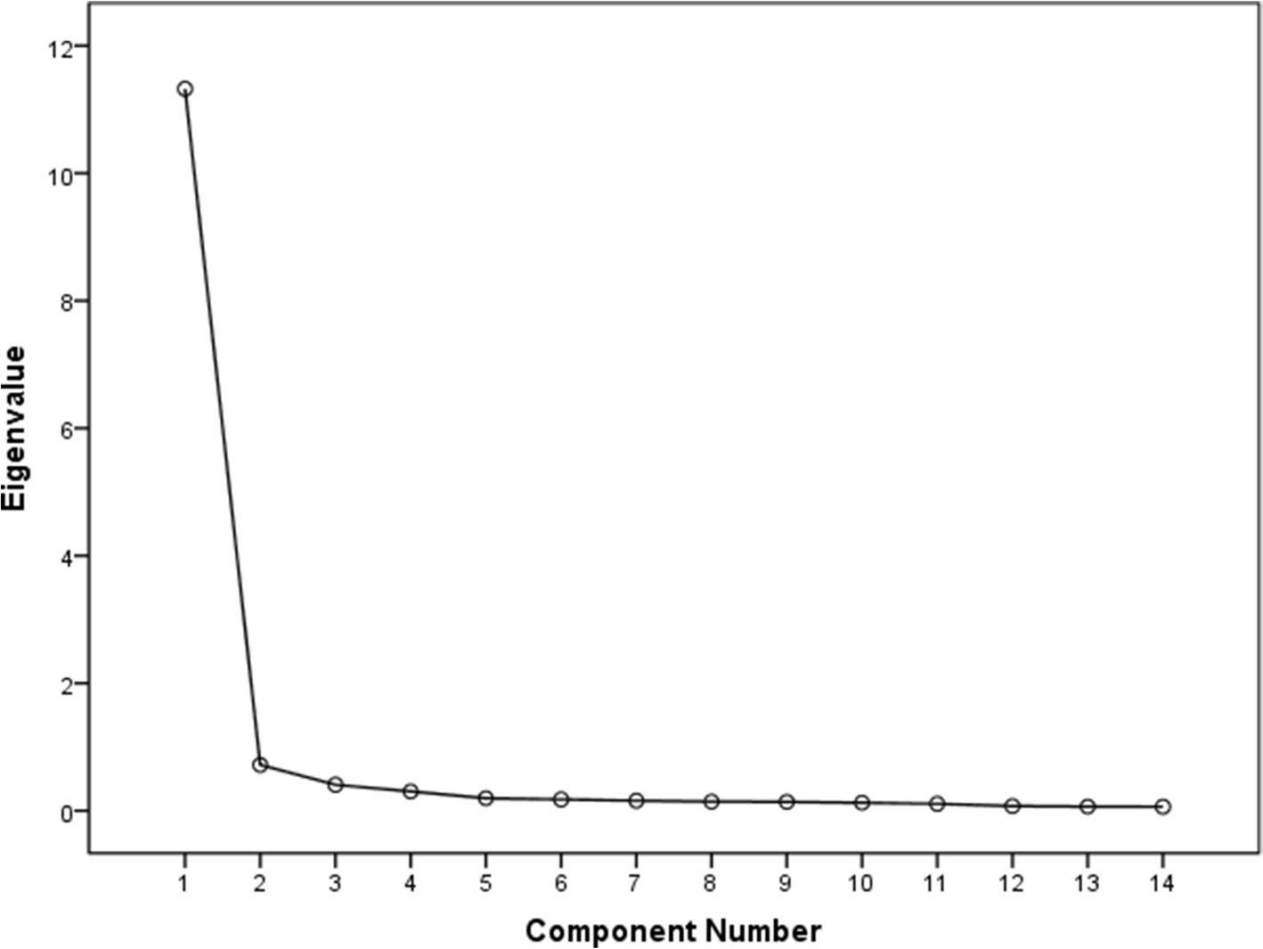
Scree plot

The results of correlation coefficient matrix showed medium and high correlations of the 14 variables (*P* < 0.05 for all of them). The results of a Bartlett test revealed that *P* < 0.05, suggesting that the correlation matrix of 14 variables is not a unit matrix, and the variables are not independent (KMO = 0.964). In summary, these variables are highly suitable for factor analysis. Based on the standard that the cumulative contribution rate of common factor should be > 85% [14], three common factors were extracted based on the Scree plot. Their commonalities on all the variables were high, indicating that they can effectively reflect the extent of variation of each variable. The factor structure after rotation was relatively clear. The first factor had large positive loads on X8, X9, X10, X11, X12, X13 and X14 and was defined as the professional ethics factor. The second factor had large positive loads on X3, X4, X5, X6 and X7 and was defined as the professional capability factor; the third factor had large positive loads on X1 and X2 and was defined as the professional emotion factor (Tables 3, 4, 5 and Fig. 1).

**Table 3:** Correlation coefficient matrix of 14 indicators of Nightingale’s nursing professionalism

	X1	X2	X3	X4	X5	X6	X7	X8	X9	X10	X11	X12	X13	X14
X1	1													
X2	0.832 < 0.001	1												
X3	0.811 < 0.001	0.771 < 0.001	1											
X4	0.796 < 0.001	0.736 < 0.001	0.916 < 0.001	1										
X5	0.798 < 0.001	0.749 < 0.001	0.859 < 0.001	0.909 < 0.001	1									
X6	0.728 < 0.001	0.683 < 0.001	0.779 < 0.001	0.771 < 0.001	0.755 < 0.001	1								
X7	0.712 < 0.001	0.671 < 0.001	0.792 < 0.001	0.784 < 0.001	0.767 < 0.001	0.844 < 0.001	1							
X8	0.790 < 0.001	0.780 < 0.001	0.835 < 0.001	0.844 < 0.001	0.840 < 0.001	0.756 < 0.001	0.795 < 0.001	1						
X9	0.751 < 0.001	0.772 < 0.001	0.754 < 0.001	0.785 < 0.001	0.775 < 0.001	0.619 < 0.001	0.634 < 0.001	0.821 < 0.001	1					
X10	0.789 < 0.001	0.802 < 0.001	0.786 < 0.001	0.805 < 0.001	0.816 < 0.001	0.686 < 0.001	0.706 < 0.001	0.835 < 0.001	0.867 < 0.001	1				
X11	0.795 < 0.001	0.785 < 0.001	0.779 < 0.001	0.803 < 0.001	0.810 < 0.001	0.679 < 0.001	0.702 < 0.001	0.843 < 0.001	0.874 < 0.001	0.924 < 0.001	1			
X12	0.751 < 0.001	0.697 < 0.001	0.833 < 0.001	0.870 < 0.001	0.860 < 0.001	0.756 < 0.001	0.770 < 0.001	0.824 < 0.001	0.775 < 0.001	0.816 < 0.001	0.815 < 0.001	1		
X13	0.780 < 0.001	0.772 < 0.001	0.793 < 0.001	0.817 < 0.001	0.821 < 0.001	0.679 < 0.001	0.695 < 0.001	0.822 < 0.001	0.880 < 0.001	0.868 < 0.001	0.875 < 0.001	0.834 < 0.001	1	
X14	0.790 < 0.001	0.757 < 0.001	0.809 < 0.001	0.837 < 0.001	0.832 < 0.001	0.694 < 0.001	0.702 < 0.001	0.847 < 0.001	0.878 < 0.001	0.860 < 0.001	0.870 < 0.001	0.854 < 0.001	0.930 < 0.001	1

**Table 4:** Results of principal component analysis

Principal component	Eigenvalue	Cumulative contribution rate (%)
1	11.325	80.890
2	0.718	86.018
3	0.409	88.937
4	0.302	91.093
5	0.197	92.499
6	0.178	93.772
7	0.157	94.895
8	0.142	95.912
9	0.138	96.900
10	0.126	97.798
11	0.108	98.570
12	0.073	99.089
13	0.065	99.555
14	0.062	100.000

**Table 5:** Factor load matrix after varimax orthogonal rotation

	F1	F2	F3
X1	0.464	0.493	0.647
X2	0.462	0.374	0.760
X3	0.520	0.690	0.349
X4	0.608	0.685	0.244
X5	0.617	0.643	0.274
X6	0.266	0.824	0.353
X7	0.319	0.828	0.289
X8	0.616	0.579	0.375
X9	0.816	0.287	0.389
X10	0.745	0.385	0.434
X11	0.764	0.375	0.417
X12	0.677	0.641	0.155
X13	0.801	0.395	0.330
X14	0.799	0.433	0.297
Contribution (%)	39.616	32.641	16.680

## Discussion

This study aims to explore the connotation of Nightingale’s nursing professionalism and the recognition of nurses and nursing students in China. A statistical analysis of the survey of different populations and their characteristic indicators revealed that nurses and nursing students in China have a consistent understanding of Nightingale’s nursing professionalism, and the average scores of all items exceeded 7, indicating that all the indices in Nightingale’s nursing professionalism are of equal significance.

Some studies proposed that nurse interns feel frustrated about the public view that nurses primarily provide ‘care’ and do not merit recognized professionalism [15]. Moreover, clinical teachers help students to acquire professional values by acting as an example in practice [16]. Therefore, it is assumed that nurses have a deep understanding of Nightingale’s nursing professionalism compared with nursing students owing to their clinical experiences. However, the survey results indicated no difference in their understandings. Changes in their understanding of professionalism after work experience could be attributed to issues in the cognition of professional role in practice.

It has been demonstrated that nurses with a negative role model have a negative impact on the development of self-confidence and professional recognition of nursing students, while those with a positive role model help nursing students to develop knowledge, skills, values and beliefs [17]. A study on the cognition of senior high school students of nurses revealed that nurse internships may have a positive impact on the nursing concept, as well as on the recruitment and retention of nurses [18]. Studies on career development demonstrated that positive role models should be established to facilitate the professional development of nursing students, while the clinical nurses, teachers and supervisors that they contact during an internship play an important role [19]. This suggests that nursing management should facilitate shaping models for nurses and nursing students to improve their professional recognition. Kelly et al*.* [20] reported that a visit to the Nightingale History Museum in UK is an effective way to enhance the professional cognition of nursing students. Therefore, it is highly significant to investigate the cognition and recognition of Nightingale and its nursing spirit by nurses and nursing students.

What exactly is Nightingale’s nursing professionalism? Based on the survey items mentioned above and the results of factor analysis, these items can be classified into professional emotion (e.g., dedication and sympathy), professional capability (e.g., keen insight, professional judgment, professional care, leadership, and innovation) and professional ethics (e.g., teamwork, exquisite skills, comfort, help, respect for life, love and dedication, prudence and responsibility). Florence Nightingale wrote in her diary that “God calls me to serve her. God’s call is for me to work hard among the poor with strong faith”. The strong belief of Florence Nightingale in nursing led her to implement nursing education and nursing reform without fear of difficulties, thus, becoming a pioneer of modern nursing [21]. Although it is not necessary for every nurse to have the same religious beliefs as Nightingale, nurses must have a persistent love for nursing and exhibit love, sympathy, and dedication to patients. This is defined as professional emotion.

Florence Nightingale also believed that love cannot replace professional training or surpass professional knowledge. Instead, deep dedication to professionalism and belief is required. She believed that nurses who do not develop professional skills via systematic learning are essentially ‘Potter and cobble’ on patients [21]. Although Humanistic care is the essence of nursing, nursing without professional knowledge as support is no more than charity. Nursing must rely on scientific knowledge and technology to enhance the value of nursing, so that they can effectively serve the public. Therefore, professional capability is an essential part of Nightingale’s nursing professionalism. In addition to advanced professional knowledge and skilled professional skills, a qualified nurse should have noble professional ethics and norms. Florence Nightingale always adhered to the character of nurses. She believed that an excellent nurse should be punctual, quiet, trustworthy, tidy and regular. Indeed, Florence Nightingale considered a good personality to be the criterion for nursing workers [21]. Studies have claimed that professional ethics are the basis of professional recognition [22]. Nursing requires nursing staff to love their careers, be prudent and responsible, and work as a team with doctors and other relevant personnel. This was Nightingale’s requirement and expectation for nursing, as well as one of the core connotations of Nightingale’s nursing professionalism in the new era. Nurses must have professional ethics, which are the necessary guarantee for the physical and mental rights of patients, and it is the third constituent element of Nightingale’s nursing professionalism. This is defined as professional ethics.

## Limitations

The major limitation of this study is the representativeness and universality of the sample. This study solely reflects the situation of China and does not involve nurses in other countries. Moreover, the survey was conducted by snowball sampling with no targeted and rigorous control design. Thus, it is impossible to compare the survey results of nurses in hospitals in different regions. Additionally, the customized questionnaire used in this study may be biased in terms of items. Therefore, it is suggested that future studies involve more samples and a qualitative interview to comprehensively and deeply understand the cognition and recognition of Nightingale’s nursing professionalism by nurses.

## Conclusions

Although the data need to be interpreted with caution owing to the limitations described above, the factor analysis suggests that the structure of Nightingale’s nursing professionalism includes professional emotion, professional ability and professional ethics, which are all essential and constitute an interactive triangular model. These three factors can only result in a high level of professionalism by interactive promotion and improvement. Moreover, the survey results indicate that nurses and nursing students have the same level of understanding of Nightingale’s nursing professionalism. They highly recognize Nightingale’s nursing professionalism, indicating that this model is still applicable and plays a guiding role for nurses in contemporary times. This suggests that nursing education and nursing managers should fully utilize this model to improve the professionalism of nurses.

## Data Availability

The data garnered during the current study and the final dataset used for statistical analysis are available from the corresponding author on reasonable request.

## References

[1] Fantahun A, Demessie A, Gebrekirstos K, Zemene A, Yetayeh G (2014). A cross sectional study on factors influencing professionalism in nursing among nurses in Mekelle Public Hospitals, North Ethiopia, 2012. BMC Nurs.

[2] Nelson S, Gordon S (2004). The rhetoric of rupture: Nursing as a practice with a history. Nurs Outlook.

[3] Wang Q, Zhu R, Duan Z (2018). The historical evolution of the regulations for the Florence Nightingale Medal. Front Nurs.

[4] Wang Q, Zhu R, Duan Z (2021). An Analysis of Past Florence Nightingale Medal Recipients: Insights Into Exceptional Nurses and the Evolution of Nursing. SAGE Open Nurs.

[5] Roxanne N (2003). Good night, Florence: with nursing in crisis, some say it's time to retire Nightingale as a symbol. Nurs Leadership.

[6] Hall RH (1968). Professionalization and Bureaucratization. Am Sociol Rev.

[7] Miller BK (1985). Just what is a professional. Nurs Success Today.

[8] Ichikawa N, Yamamoto-mitani N, Takai Y, Tanaka M, Takemura Y (2020). Understanding and measuring nurses' professionalism: Development and validation of the Nurses' Professionalism Inventory. J Nurs Manag.

[9] Hwang JI, Lou F, Han SS, Cao F, Li P (2010). Professionalism: the major factor influencing job satisfaction among Korean and Chinese nurses. Int Nurs Rev.

[10] Koo M, Lin SC (2016). The image of nursing: A glimpse of the Internet. Jpn J Nurs Sci.

[11] Cingel M, Brouwer J (2021). What makes a nurse today? A debate on the nursing professional identity and its need for change. Nurs Philos.

[12] Lynette C, Janie D, Hunter B (2019). Re-engaging Concepts of Professionalism to Inform Regulatory Practices in Nursing. J Nurs Regul.

[13] Lynn MR (1986). Determination and quantification of content validity. Nurs Res.

[14] Rosenblad A . A Step-by-Step Approach to Using SAS® for Factor Analysis and Structural Equation Modelling, Second Edition[J]. Int Statistical Rev. 2015;83(2):325–26.

[15] Keeling J, Templeman J (2013). An exploratory study: Student nurses' perceptions of professionalism. Nurse Educ Pract.

[16] Brown J, Stevens J, Kermode S (2012). Supporting student nurse professionalisation: The role of the clinical teacher. Nurse Educ Today.

[17] Tesk G, Boz I (2019). I try to act like a nurse: A phenomenological qualitative study. Nurse Educ Pract.

[18] Porter G, Edwards PB, Granger BB (2009). Stagnant perceptions of nursing among high school students: results of a shadowing intervention study. J Profess Nurs.

[19] Felstead IS, Springett K (2016). An exploration of role model influence on adult nursing students' professional development: A phenomenological research study. Nurse Educ Today.

[20] Kelly J, Watson R, Watson J, Needham M, Driscoll LO (2017). Studying the old masters of nursing: A critical student experience for developing nursing identity. Nurse Educ Pract.

[21] Barritt ER (2010). Florence Nightingale's Values and Modern Nursing Education. Nurs Forum.

[22] Crigger N, Godfrey N (2014). From the Inside Out: A New Approach to Teaching Professional Identity Formation and Professional Ethics. J Prof Nurs.

